# New Dosing Interval and Schedule for the Bexsero MenB-4C Vaccine: Updated Recommendations of the Advisory Committee on Immunization Practices — United States, October 2024

**DOI:** 10.15585/mmwr.mm7349a3

**Published:** 2024-12-12

**Authors:** Sarah Schillie, Jamie Loehr, Wilbur H. Chen, Charlotte A. Moser, Gabrielle Cooper, Cheryl Isenhour, Lucy A. McNamara

**Affiliations:** ^1^Division of Bacterial Diseases, National Center for Immunization and Respiratory Diseases, CDC; ^2^Cayuga Family Medicine, Ithaca, New York; ^3^Center for Vaccine Development and Global Health, University of Maryland School of Medicine, Baltimore, Maryland; ^4^Children’s Hospital of Philadelphia, Philadelphia, Pennsylvania.

**Summary**

**What is already known about this topic?**

Meningococcal disease is a life-threatening invasive infection caused by *Neisseria meningitidis*. MenB-4C (Bexsero, GSK), one of two licensed meningococcal serogroup B vaccines, protects against serogroup B *N. meningitidis* and is licensed for persons aged 10–25 years.

**What is added by this report?**

On October 24, 2024, the Advisory Committee on Immunization Practices (ACIP) updated its recommendations for MenB-4C to align the dosing interval and schedule with the new Food and Drug Administration (FDA) label and harmonize with recommendations for MenB-FHbp (Trumenba, Pfizer, Inc.) vaccine. ACIP now recommends MenB-4C as a 2-dose series with doses administered at intervals of 0 and 6 months for healthy adolescents and young adults aged 16–23 years based on shared clinical decision-making and as a 3-dose series with doses administered at 0, 1–2, and 6 months for persons aged ≥10 years at increased risk.

**What are the implications for public health practice?**

The new MenB-4C dosing interval and schedule improves immune protection. ACIP recommendations for the MenB-4C dosing interval and schedule are now aligned with the updated FDA label and are harmonized with ACIP recommendations for use of MenB-FHbp.

## Abstract

Two meningococcal serogroup B vaccines are licensed for use in the United States. In August 2024, the Food and Drug Administration (FDA) changed the label for the meningococcal serogroup B MenB-4C vaccine (Bexsero) from a 2-dose schedule (intervals of 0 and ≥1 month) to a 2-dose schedule (0 and 6 months) and added a 3-dose schedule (0, 1–2, and 6 months), based on new immunogenicity data. On October 24, 2024, the Advisory Committee on Immunization Practices (ACIP) voted to update its recommendations for the MenB-4C dosing interval and schedule to align with the new FDA label. ACIP recommends extending the interval for the 2-dose series of MenB-4C from 0 and ≥1 month to 0 and 6 months for healthy adolescents and young adults aged 16–23 years based on shared clinical decision-making and has added a recommendation for a 3-dose series with doses administered at 0, 1–2, and 6 months for persons aged ≥10 years at increased risk. The updated ACIP recommendations for MenB-4C align with existing ACIP recommendations for the other FDA-licensed meningococcal serogroup B vaccine, MenB-FHbp (Trumenba).

## Introduction

Vaccination against serogroup B meningococcal disease is recommended by the Advisory Committee on Immunization Practices (ACIP) for adolescents and young adults aged 16–23 years, based on shared clinical decision-making (https://www.cdc.gov/vaccines/hcp/admin/downloads/ISD-job-aid-SCDM-mening-b-shared-clinical-decision-making.pdf); and for persons aged ≥10 years who are at increased risk for serogroup B meningococcal disease (i.e., persons with anatomic or functional asplenia, complement component deficiencies, or complement inhibitor use; microbiologists routinely exposed to *Neisseria meningitidis* isolates; and persons at increased risk during an outbreak). Shared clinical decision-making recommendations are intended to be flexible and are guided by a decision-making process between the health care provider and the patient or the patient’s parent or guardian. Considerations for shared clinical decision-making for vaccine administration might include the serious nature of meningococcal infections, the low number of serogroup B meningococcal disease cases, the estimated relatively short duration of protection from MenB vaccines (antibody waning within 1–2 years after completion of the primary series), and the increased risk among college students, especially those who are freshmen, attend a 4-year university, live in on-campus housing, or participate in sororities and fraternities. Two serogroup B meningococcal vaccines (MenB-FHbp [Trumenba, Pfizer, Inc.] and MenB-4C [Bexsero, GSK]) and one serogroup ABCWY pentavalent meningococcal (MenABCWY) vaccine (MenACWY-TT/MenB-FHbp [Penbraya, Pfizer Inc.]) are currently licensed for use in persons aged 10–25 years in the United States (*1*,*2*). Primary series MenB vaccination in persons aged ≥26 years and booster vaccination in persons at increased risk for meningococcal disease are not licensed in the United States and are considered off-label ACIP recommendations.

ACIP recommends MenB-FHbp vaccine as a 2-dose series (at intervals of 0 and 6 months) for adolescents and young adults and a 3-dose series (at 0, 1–2, and 6 months) for those at increased risk. Previously, ACIP recommended MenB-4C vaccine as a 2-dose series (at 0 and ≥1 month) for adolescents and young adults and those at increased risk, consistent with FDA licensure (*1*). In August 2024, FDA changed the label for MenB-4C from a 2-dose schedule (0 and ≥1 month) to a 2-dose schedule (0 and 6 months) and added a 3-dose schedule (0, 1–2, and 6 months) (*3*). These changes were prompted by new immunogenicity data and were not due to safety concerns (*4*). This report summarizes evidence considered for the MenB-4C dosing interval and schedule changes and provides clinical guidance for the use of MenB vaccines. This report only updates recommendations for the dosing interval and schedule for MenB-4C; other previously published meningococcal vaccination guidance remains unchanged (*1*,*2*).

## Methods

### Data Source and Assessment of Immunogenicity

During August–October 2024, the ACIP Meningococcal Vaccines Work Group (Work Group) held bimonthly conference calls to review meningococcal disease epidemiology and evidence regarding the dosing interval and schedule for MenB-4C in persons for whom MenB vaccination is recommended. The Evidence to Recommendations framework was used to guide deliberations; the Work Group considered the importance of meningococcal disease as a public health problem, benefits and harms, values of the target population, acceptability, resource use, equity, and feasibility (*5*). The Work Group and ACIP reviewed comparative data on the immunogenicity and safety of MenB-4C when administered using different dosing intervals and schedules. Data from five clinical trials (four published and one unpublished) are included in the updated package insert; data from three trials (two published and one unpublished) were considered in the assessment of immunogenicity.

## Summary of Evidence

### Immunogenicity

The percentage of participants achieving seroresponse was compared across four antigen indicator strains using an assay measuring serum bactericidal activity with an exogenous source of human complement (human serum bactericidal activity [hSBA]). Seroresponse was defined as a postvaccination hSBA titer at least fourfold the limit of detection (LOD) or at least the lower limit of quantitation (LLOQ), whichever is greater, for participants with prevaccination hSBA titer less than LOD, a postvaccination hSBA titer at least fourfold the LLOQ for participants with prevaccination hSBA titer at least meeting LOD and less than LLOQ, and a postvaccination hSBA at least fourfold the prevaccination hSBA titer for participants with prevaccination hSBA titer at least meeting LLOQ. Immunogenicity data were based on 1,803 persons aged 10–25 years who received at least 1 dose of MenB-4C; approximately 30% of vaccine recipients were from the United States. The proportion of persons achieving seroresponse to four antigen indicator strains ranged from 54%–97% after dose 2 of a 0-, 2-, and 6-month schedule to 57%–95% and 57%–99% after dose 2 of a 0- and 6-month schedule and dose 3 of a 0-, 2-, and 6-month schedule, respectively (*3*,*4*) (Table). 

**TABLE:** Number and percentage of persons aged 10–25 years achieving seroresponse to antigen indicator strains* 1 month after receipt of dose 2 (0- and 6-month schedule), and doses 2 and 3 (0-, 2-, and 6-month schedule) of MenB-4C (Bexsero) vaccine (N = 1,803) — United States, Australia, Canada, Czechia, Estonia, Finland, and Turkey, 2020–2022

Antigen indicator strain*	0- and 6-mo schedule		0-, 2-, and 6-mo schedule			
	Dose 2		Dose 2		Dose 3	
	No.	% (95% CI)	No.	% (95% CI)	No.	% (95% CI)
fHbp	699	78 (74–81)	739	67 (64–71)	679	81 (78–84)
NadA	700	95 (93–97)	738	97 (95–98)	679	99 (98–99)
NHBA	704	69 (66–72)	739	58 (55–62)	685	67 (63–70)
OMV	664	57 (53–61)	724	54 (50–57)	637	57 (53–60)

**Abbreviations**: fHbp = factor H binding protein; NadA = neisserial adhesin A; NHBA = neisserial heparin binding antigen; OMV = outer membrane vesicle.

* Bacterial antigenic strains for which immune response was assessed.

### Safety

MenB-4C safety data have been reported previously (*1*). The most common solicited adverse reactions after receipt of MenB-4C were pain at the injection site (≥87% of recipients), fatigue (≥45%), and headache (≥37%). Adverse reactions occurred with similar frequency after both doses in the 0- and 6-month schedule and each dose in the 0-, 2-, and 6-month schedule (*3*,*4*).

### Evidence to Recommendations Framework Domains

An extended interval between doses might be associated with reduced series completion and might disproportionately affect some populations. During 2022, among adolescents initiating MenB vaccination who were not eligible for the Vaccines for Children (VFC) program,* 49.6% of MenB-4C recipients and 35.5% of MenB-FHbp recipients completed their series by age 17 years. Among those initiating MenB vaccination who were eligible for the VFC program, 51.4% and 16.2% completed their MenB-4C and MenB-FHbp series, respectively, by age 17 years. Among commercially insured adolescents in the Merative MarketScan Commercial Claims and Encounters database,^†^ 67% and 60% of those initiating vaccination completed their MenB-4C and MenB-FHbp series, respectively, by age 19 years during 2017–2023; series completion by age 19 years could not be assessed for the VFC-eligible population because this population was not ascertained in the database. The Work Group deliberations included potential detrimental effects on equity with extended dosing intervals and favorable effects regarding feasibility associated with harmonization of MenB-4C and MenB-FHbp recommendations (*4*).

## ACIP Recommendations

These recommendations apply to use of the 2- and 3-dose schedules of MenB-4C and supersede previous ACIP recommendations for use of MenB-4C published in 2020 (*1*). Recommendations regarding use of MenB-FHbp and MenACWY-TT/MenB-FHbp, as well as recommendations for booster doses, are unchanged (*1*,*2*).

### Adolescents and Young Adults Aged 16–23 Years (Shared Clinical Decision-Making Recommendation)

ACIP recommends that MenB-4C be administered to healthy adolescents and young adults aged 16–23 years as a 2-dose series at 0 and 6 months for the prevention of serogroup B meningococcal disease, based on shared clinical decision-making (Box). Shared clinical decision-making recommendations are not recommended for everyone in an age or risk group but are individually based and guided by a decision process between the health care provider and the patient or the patient’s parent or guardian.

BOX MenB-4C vaccination recommendations* — Advisory Committee on Immunization Practices, United States, 2024

**Adolescents and young adults aged 16–23 years (shared clinical decision-making recommendation)**

- MenB-4C is recommended for healthy adolescents and young adults aged 16–23 years.
- Administer as a 2-dose series at intervals of 0 and 6 months, based on shared clinical decision-making.
- All doses in a series, in addition to booster doses, should be from the same manufacturer.

**Persons aged ≥10 years at increased risk for serogroup B meningococcal disease**

- MenB-4C is recommended for persons aged ≥10 years who are at increased risk for serogroup B meningococcal disease:
  - Persons with anatomic or functional asplenia
  - Persons with complement component deficiencies
  - Persons using a complement inhibitor
  - Microbiologists routinely exposed to *Neisseria meningitidis* isolates
  - Persons at increased risk during an outbreak
- Administer MenB-4C as a 3-dose series at 0, 1–2, and 6 months.
- All doses in a series, in addition to booster doses, should be from the same manufacturer.

* https://www.fda.gov/media/90996/download

### Persons Aged ≥10 Years at Increased Risk for Serogroup B Meningococcal Disease

ACIP recommends that MenB-4C be administered as a 3-dose series at 0, 1–2, and 6 months to persons aged ≥10 years who are at increased risk for serogroup B meningococcal disease (i.e., persons with anatomic or functional asplenia, complement component deficiencies, or complement inhibitor use; microbiologists routinely exposed to *N. meningitidis* isolates; and persons at increased risk during an outbreak).

## CDC Guidance for Use

### Interchangeability of Vaccine Products

Two manufacturers provide three MenB vaccine products (*1*,*2*) that are licensed and available for use in the United States. MenB vaccines from different manufacturers are not interchangeable; all doses in a series, as well as booster doses, should be from the same manufacturer. If doses from both manufacturers have been administered to the same patient, the patient should receive a complete series of either manufacturer’s product without counting doses of the other manufacturer as valid. The next dose of the selected manufacturer should be administered no sooner than the recommended interval after the previous dose from the same manufacturer and ≥4 weeks after the most recent dose (from either manufacturer) was administered (*1*).

### Dosing Interval and Schedule

There is no recommendation to administer additional doses to persons vaccinated with MenB-4C before October 24, 2024, using a 0- and 1-month dosing schedule. Persons who have completed a MenB vaccine primary series and who remain or become at increased risk for invasive meningococcal disease are recommended to receive booster vaccination. Booster doses need to be from the same manufacturer used for doses in the primary series.

When administering the 2-dose series (e.g., for healthy adolescents) of MenB-4C (Bexsero) or MenB-FHbp (Trumenba), the 2 doses should be separated by 6 months. If the second dose is administered <6 months after the first dose, a third dose should be administered ≥4 months after the second dose. A second dose administered at an interval >6 months after the first dose is valid and does not need to be repeated (*1*).

When administering the 3-dose series (e.g., for persons aged ≥10 years at increased risk for MenB infection) of MenB-4C (Bexsero) or MenB-FHbp (Trumenba), a third dose is not needed if the second dose was administered ≥6 months after the first dose. If the third dose is administered <4 months after the second dose, the dose should be repeated ≥4 months after the last dose, unless the third dose was administered ≥6 months after the first dose (*1*,*3*,*6*).

Persons receiving a MenB vaccine based on shared clinical decision-making who desire more rapid protection against serogroup B (e.g., students initiating vaccination <6 months before college entry) may receive the 3-dose series (0, 1–2, and 6 months) to optimize rapid protection. This guidance applies to both MenB-4C and MenB-FHbp. When deciding timing of vaccination, providers may consider that VFC program eligibility ends at age 19 years.

### Persons Taking Complement Inhibitors

Persons on complement inhibitor therapy likely remain at substantially increased risk for meningococcal disease, even if they are fully vaccinated or taking antimicrobial prophylaxis. Although evidence suggests that vaccination might not adequately prevent meningococcal infections among persons with certain complement deficiencies or those using a complement inhibitor, these persons should continue to be vaccinated according to recommendations because of the potential for benefit among persons at high risk for infection (*1*).

Persons not up to date with meningococcal vaccinations for whom urgent complement inhibitor therapy is indicated should be provided antimicrobial prophylaxis (*1*,*3*). Few data are available to guide decision-making regarding the optimal duration of antimicrobial prophylaxis; therefore, the duration of prophylaxis should be determined based on clinical judgment. Providers could consider treating patients with antimicrobial prophylaxis for the duration of complement inhibitor treatment.

### Reporting of Vaccine Adverse Events

Adverse events after vaccination should be reported to the Vaccine Adverse Event Reporting System. Reporting is encouraged for any clinically significant adverse event, even when a causal association between the vaccine and the event is uncertain, as well as for vaccination errors. Additional information is available at https://vaers.hhs.gov or by telephone at 1-800-822-7967.

## Footnotes

* VFC provides vaccines at no cost to children and adolescents aged ≤18 years who are uninsured, underinsured, Medicaid-eligible, or American Indian or Alaska Native. https://www.cdc.gov/vaccines-for-children/about/index.html

†
https://www.merative.com/content/dam/merative/documents/brief/marketscan-explainer-general.pdf
